# Screening and prioritization of nano- and microplastic particle toxicity studies for evaluating human health risks – development and application of a toxicity study assessment tool

**DOI:** 10.1186/s43591-021-00023-x

**Published:** 2022-01-14

**Authors:** Todd Gouin, Robert Ellis-Hutchings, Leah M. Thornton Hampton, Christine L. Lemieux, Stephanie L. Wright

**Affiliations:** 1 TG Environmental Research, Sharnbrook, Bedfordshire, UK; 2 Toxicology and Environmental Research & Consulting, The Dow Chemical Company, Midland, MI 48673 USA; 3 Department of Toxicology, Southern California Coastal Water Research Project, Costa Mesa, CA USA; 4 Air Quality and Risk Assessment Division, Water and Air Quality Bureau, Health Canada, Ottawa, ON K1A 0K9 Canada; 5 Environmental Research Group, School of Public Health, Imperial College London, Sir Michael Uren Hub, 86 Wood Lane, London, W12 0BZ UK

**Keywords:** Microplastic, Quality assurance/quality control, Human health, Risk assessment

## Abstract

Concern regarding the human health implications that exposure to nano- and microplastic particles (NMPs) potentially represents is increasing. While there have been several years of research reporting on the ecotoxicological effects of NMPs, human health toxicology studies have only recently emerged. The available human health hazard data are thus limited, with potential concern regarding the relevance and reliability for understanding the potential human health implications. In this study we develop and apply a NMP toxicity screening assessment tool (NMP-TSAT) for evaluating human health effects studies against a suite of quality assurance and quality control (QA/QC) criteria for both in vivo and in vitro studies. A total of 74 studies representing either inhalation or oral exposure pathways were identified and evaluated. Assessment categories include particle characterization, experimental design, and applicability for risk assessment; with critical and non-critical criteria organized to allow screening and prioritization. It is observed that the majority of studies evaluated using the NMP-TSAT have been performed on monodisperse particles, predominately spheres (≈60%), consisting of polystyrene (≈46%). The majority of studies have tested particles < 5 μm, with a minimal particle size of 10 nm and a maximum particle size of about 200 μm. The total assessment score (TAS) possible for in vivo studies is 52, whereas for in vitro studies it is 46, which is based on receiving a maximum score of 2 against 26 and 23 criteria, respectively. The evaluated TAS ranged from between 12 and 44 and 16–34, for in vivo and in vitro studies, respectively. Given the challenges associated with prioritizing studies based on ranking them according to their TAS we propose a Tiered approach, whereby studies are initially screened based on how they score against various critical criteria, which have been defined for their relevance for assessing the hazards and risks for human health. In this instance, studies that score a minimum of ‘1’ against each of the critical criteria, regardless of how they rank according to their TAS, are prioritized as part of a Tier 1 screening and prioritization phase, which would then be followed by an expert evaluation, representing a Tier 2 level of assessment. Using this approach we identify 10 oral ingestion and 2 inhalation studies that score at least 1 against all critical criteria. Lastly, several key observations for strengthening future effects studies are identified, these include a need for the generation and access to standard reference materials representative of human exposure to NMPs for use in toxicity test systems and/or the improved characterization and verification of test particle characteristics, and the adoption of study design guidance, such as recommended by OECD, when conducting either in vivo inhalation or oral ingestion toxicity tests.

**Supplementary Information:**

The online version contains supplementary material available at 10.1186/s43591-021-00023-x.

## Introduction

The relevance and reliability of data generated from effects studies are understood to represent the two fundamental elements to consider when assessing the adequacy of data for use in predicting whether chemical and non-chemical stressors represent an environmental and/or human health risk. There have been several approaches proposed and applied for assessing the relevance and reliability of effects data for a range of stressors [1–9] and which have been reviewed by Moermond et al. [10]. An important observation is that the application of a specific method should ensure that the assessment criteria are fit-for-purpose, which may vary depending on the purpose and intended use of the data [10]. Fundamentally, the evaluation of the relevance and reliability of effect studies should be conducted following a systematic and transparent method that utilizes expert judgement [10–13].

Recent efforts to evaluate the environmental and human health risk of nano- and microplastic particles (NMPs) have encountered challenges due to limited information towards characterizing and quantifying exposure as well as the lack of availability of standardized tests aimed at quantifying adverse effects for use in risk assessment [14–20]. Nevertheless, there is evidence that exposure to NMPs is can occur in the environment [21–24], however the characterization and quantification of human exposure to NMPs remains an important data need. Only a limited number of studies reporting exposure data for NMPs have been obtained, mostly derived using non-standard methods and for particle sizes generally > 10 μm, with no data currently sufficient to characterize and quantify exposure to nanoplastic particles [23–28]. At the same time there is a significant increase in the number of publications investigating the potential adverse health effects of NMPs on the environment and human health [18]. Recently, de Ruijter et al. [3] evaluated the relevance and reliability of 105 ecotoxicological effects studies for NMPs against 20 quality criteria and observed a lack of consistency across all studies towards quality assurance. They further observed a tendency for studies to speculate mechanisms that are poorly supported by the design and reporting of the data in the study [3].

Despite the continuing increase in the number of publications investigating the effects of NMPs in human and mammalian models, an assessment similar to that of de Ruijter et al. [3] regarding the quality of the data for informing the human health risk assessment is lacking. Assessing both the exposure and hazards of NMPs for human health represents a substantive challenge for regulatory authorities, who are currently witnessing an exponential rate of increase in publications in the peer-review literature, and which represents a significant and non-trivial resource issue towards keeping up with the latest scientific advances. This is further exacerbated by research published in the peer-review literature that reports adverse effects of NMPs based on adapting existing test systems or developing novel approaches towards assessing their potential human health implications. Given that the availability of standard test systems appropriate for regulatory assessment and mechanistic understanding related to a toxicological mode-of-action or an adverse outcome pathway is currently lacking for NMPs [18], it is important that fundamental research retain flexibility to explore and strengthen scientific understanding. It is thus anticipated that greater confidence in the hazard assessment of NMPs, for instance, will greatly benefit from scientific ingenuity aimed at characterizing and quantifying mechanistic understanding regarding the relationship between the physicochemical properties of the particles and an observed adverse effect threshold of clinical relevance, particularly from studies that include strong elements of quality assurance and quality control (QA/QC) [19].

NMPs in the environment are understood to be comprised of a complex heterogenous mixture of properties, which can be described based on probabilistic distribution functions [29]. Additionally, it is observed that NMPs can contain complex mixtures of chemicals, either sorbed from the environment or intentionally added in the original plastic product [24, 30–32]. While individual chemicals used in plastic products have undergone risk assessment, particularly in relation to food contact materials [33], potential releases into the environment due to littering or in relation to the degradation and fragmentation of plastic debris have not been robustly assessed. Differentiating the potential effects and modes-of-action of particles from those of complex mixtures of chemicals, and the interactions between chemical and non-chemical effects, can result in a near impossible task of needing to test every possible combination [31, 34]. Thus, it may be beneficial to consider the utility of a strategic intelligent testing strategy [35, 36], aimed at strengthening our ability to holistically assess the risks associated with environmentally relevant exposures to NMPs [20]. Addressing QA/QC concerns would represent an important element to any future testing strategies.

Recognizing that there currently exists no standard test protocols for characterizing and quantifying adverse effects of NMPs and that regulators tend towards preferring the use of data generated from standard tests over non-standard tests [1], combined with various uncertainties related to the reliability and relevance of studies published in the peer-reviewed literature in relation to NMPs [3, 14, 27, 37], efforts to critically review current QA/QC practices and provide guidance for strengthening the use of non-standard data are needed [19]. Thus, in order to facilitate progress towards science-based human health risk assessment for NMPs, an important way forward is to enable increased use of all available data identified as fit-for-purpose. Evaluating data that are fit-for-purpose in the context of human health risk assessment requires the ability to screen and prioritize studies accordingly. There is currently no such method available for NMPs. The overall aim of this study is to improve the scientific basis for evaluating the potential human health implications associated with exposure to NMPs through the development and application of evaluation criteria, which we suggest can be used by risk assessors and researchers. This study describes the development and application of a toxicity screening and assessment tool for NMPs, which can be used as a tier 1 screening and prioritization tool within a tiered approach for evaluating the relevance and reliability of toxicity studies for NMPs. The approach taken builds upon other tools that have been developed and applied to systematically screen and prioritize studies reporting environmental and human health effects for various chemical and particle stressors. Studies that move to the higher tier 2 evaluation are subject to further scrutiny by toxicologists with specific expertise capable of evaluating specific endpoints [38].

## Method

The adoption and adaptation of methods similar to existing quality evaluation tools, such as Klimisch and the criteria for reporting and evaluating ecotoxicity data (CRED), have been developed and applied to assess the quality of data emerging from studies reporting on exposure concentrations of MPs in biota, surface and drinking water, air, and ecotoxicity studies [3, 16, 27, 37, 39]. Currently, a similar evaluation method for evaluating the QA/QC components of NMPs effect studies related to human health implications is lacking.

**Fig. 1:**
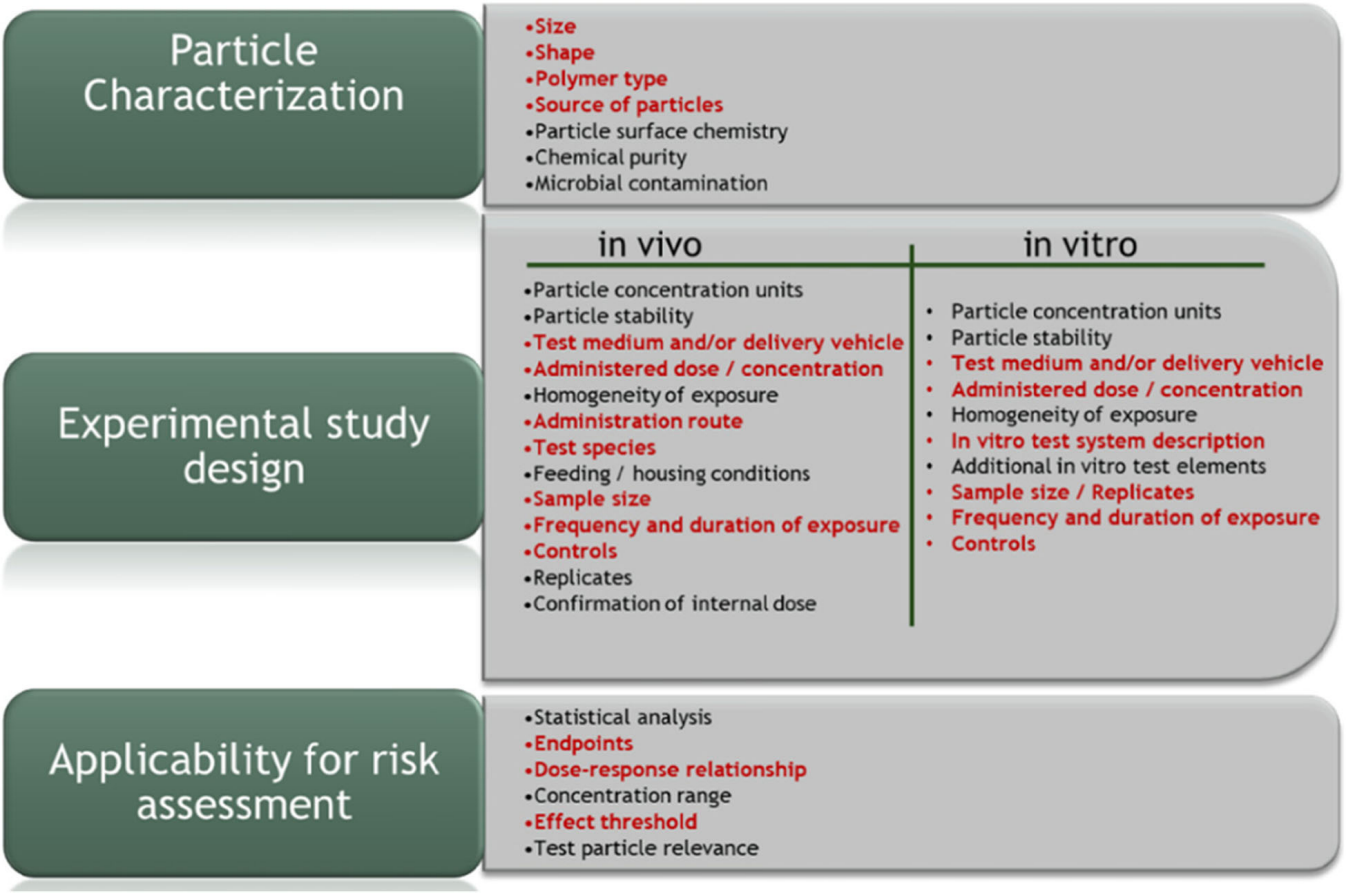
Schematic summary of the approach used to evaluate in vivo and in vitro effects studies for use in assessing human health risks related to exposure to nano- and microplastic particles. All criteria have equal weight, whereby studies receiving non-zero scores against all criteria would ideally represent studies that should be prioritized for risk assessment. Due to an absence of any study receiving non-zero scores against all criteria, however, the criteria highlighted in red represent the minimal information required for identifying a study as fit-for-purpose in the context of deriving a human health threshold value by participants of the Southern California Coastal Water Research Project (SCCWRP) Health Effects Workshop (https://www.sccwrp.org/about/research-areas/additional-research-areas/trash-pollution/microplastics-health-effects-webinar-series/. The ‘red’ criteria can be modified depending on the screening and prioritization purpose and should not be misinterpreted as representing criteria that are critical for determining the reliability and relevance of a study. Tables S1 and S2 provide guidance related to scoring each of the criteria

The approach described herein combines methods and criteria that have been used in both the development of the ToxRtool [8], developed by the Joint Research Centre (JRC), with particular emphasis on modifications made to evaluate toxicity studies on engineered nanomaterials developed as part of the European Union FP-7 GUIDEnano project [9], and the evaluation criteria used by de Ruijter et al. [3], which is used to evaluate ecotoxicity studies for MPs. The approach adopted here is thus intended to enable an evaluation of both in vivo mammalian effects studies and in vitro bioassays with relevance towards assessing the potential human health implications of exposure to MPs. A consistent element adopted in the tools presented by both Fernández-Cruz et al. [9] and de Ruijter et al. [3] is the emphasis towards evaluating three separate components of a study. These include an evaluation of how well authors address and report QA/QC criteria associated with [1] particle characterization, [2] study design and [3] relevance of the study for use in risk assessment (Fig. 1). The reporting of various QA/QC criteria aligned to these three areas is understood to represent a critical source of information needed by experts evaluating the relevance and reliability of a study for use in establishing and providing guidance in the context of understanding mechanisms of toxicological action, human health exposure threshold values and in assessing risk [25, 26]. Consistent with the approach and recommendations made by Hermsen et al. [39], Koelmans et al. [37] and de Ruijter et al. [3], each criterion in the current approach is assigned a score of adequate [2], adequate with restrictions [1] or inadequate (0) with respect to how well a study addresses the QA/QC criterion. While all criteria are assigned equal weighting, it is also possible to enable the evaluation of scores to be screened by identifying some criteria as representing particularly important elements to help in prioritizing studies that might be used for a specific purpose [40]. For instance, in order to screen and prioritize studies for use in determining a point of departure (POD) for use in human health risk assessment, only those studies that report dose-response results that are sufficient to derive either a no-observable adverse effect level (NOAEL), lowest-observable adverse effect level (LOAEL) or benchmark dose (BMD) would be perceived as fit-for-purpose. It is important to note, however, that studies that do not include data sufficient to derive a POD should not necessarily be perceived to be of lower quality, rather, they are not fit-for-purpose for deriving a POD for quantitative human health risk assessment. A summary of the specific guidance for evaluating each of the criteria for in vivo and in vitro studies is provided in Tables S1 and S2 of the Supplementary Information. The NMP-toxicity study assessment tool (TSAT) is freely available for download (https://tger.co.uk/research).

### Particle characterization

Adequate characterization of the properties of NMPs used in studies assessing their potential human health implications represents a fundamental and critical component of QA/QC study evaluation [3, 9, 17, 26]. Because of the heterogeneous nature of NMPs in the environment, with varying shapes, sizes and polymer compositions, relating the variability of these properties to differences in observed toxicological hazards (such as differences in dose-response, adverse versus adaptive effects and mechanisms of action) should represent an important consideration when designing toxicity studies.

Particle size represents a critical property that is understood to influence effects [9, 26]. The size of particles can influence the mechanism for potential translocation (e.g., smaller particles becoming systemically available by crossing epithelial and endothelial barriers, whereas larger particles can be engulfed by macrophages and cleared to lymph nodes or the lymphatic system). With respect to inhalation studies, Ferin et al. [41], for instance, observed that particles in the size range of 20-30 nm more easily penetrated the interstitial space in the lung than those 200-500 nm in size following intratracheal instillation. In their study of nano-polystyrene particles (average diameter of 56 nm and 202 nm), Chen et al. [42] dosed male Sprague-Dawley rats with 0.6 mg of radioiodinated polystyrene particles. The results suggest that only a small fraction of intratracheally-instilled particles reach the lung and can pass rapidly into systemic circulation, but that translocation is markedly increased following an infusion of lipopolysaccharides (LPS), used to induce pulmonary inflammation [42]. The use of radiolabelled particles, in this instance, helps to better characterize and quantify the bioavailability and transport of particles within biological tissues and thus greatly strengthens the potential for mechanistic understanding of processes influencing the potential for translocation. Investigating the relationship between physiological processes (e.g., pulmonary inflammation) and the physicochemical properties of the particles (e.g., size and shape) can greatly advance our overall understanding of the potential human health implications of exposure to NMPs.

Shape, for instance, represents an important factor where there is a need to differentiate between fragments, spheres and fibres. With respect to fibres, additional consideration should be given regarding the fibre-toxicological paradigm, which is also relevant for synthetic polymer fibres [13]. Adverse effects for fibres are known be influenced by their aspect ratio, length and fibre flexural rigidity, factors which influence their relative biopersistence. While the length of a fibre might be used to represent its size, the relationship between length and fibre diameter should also be considered. Rigidity is an intrinsic fibre property and can determined by the bending modulus and the diameter. Biopersistence is determined by water solubility and durability in biological media like the phagolysosomal fluid. Studies reporting adverse effects for fibres, consequently, should consider each of these additional parameters as part of their reporting on particle characteristics.

For studies investigating effects associated with the oral ingestion of NMPs, systemic uptake is understood to occur only when NMPs are absorbed by the intestinal epithelium, pass the liver, and are distributed via the blood stream throughout the entire body. Carr et al. [43], for instance, reported that < 0.32% of 2 μm latex particles were absorbed by mice. EFSA [44] have concluded that particles > 150 μm are unlikely to be absorbed, but that these larger size particles can potentially induce a local effect on the intestinal epithelium. Particles < 1.5 μm can cross the epithelium and may induce systemic toxicity at distal sites [45]. From in vitro studies, using models of the intestinal barrier, Walczak et al. [46] observed that size was a major determinant for the translocation of nano-sized particles (NPs), with up to 7.8% absorption observed for 50 nm NPs compared to only 0.8% for 100 nm NPs. Surface charge and chemistry were also observed to represent influencing factors. A comparative in vivo rat study from the same group using the same nano-sized polystyrene NPs, yielded a particle uptake in kidney, heart, stomach wall and small intestine wall, summed across all organs, of up to 1.7%, which was lower than that observed in their in vitro study [47].

Given observations that have implicated each of the properties of size, shape and surface chemistry as influencing factors with respect to various biokinetic processes, we specifically emphasize the need for studies to characterize and quantify each of these properties (Fig. 1). Ideally, each of these properties should be measured and quantified by research groups prior to conducting their effects study. While data summarized by suppliers can often provide an adequate level of information, the information can also be ambiguous and insufficient regarding the actual particle size distribution, surface charge (especially in the exposure media), and does not often include images to confirm the shape of the particles used in the study. Limited understanding of the actual characteristics of the particles tested can result in uncertainty regarding how the properties of the particles may have influenced test results. Failing to report or only providing limited details in relation to the other criteria summarized for particle characterization in Fig. 1, represent other factors that can also influence uncertainties regarding the reliability and relevance of an effects study. For instance, reporting only the source (i.e., supplier) of the particles may not necessarily provide sufficient information regarding how the particles were produced. The bottom-down production of NMPs may include various processes, such as milling, cutting, grinding, etc., which may influence size distributions, shapes or which may introduce potential confounding contaminants and alter surface chemistry [48]. Taking steps to verify the polymer composition of the particles and any actions taken to either clean the particles of any potential contaminants and/or measure the potential contamination from chemical and microbial pathogens, represent important QA/QC practices that can result in greater confidence when attempting to interpret, communicate and apply dose-response relationships attributed to the NMPs, especially in the context of evaluating potential human health implications [17].

### Experimental design

The NMP-TSAT is designed to evaluate both in vitro studies that assess human toxicity endpoints as well as mammalian in vivo studies that evaluate potential human health implications resulting from inhalation or oral exposure to NMPs. Studies that target both inhalation and oral exposure routes can be evaluated. The criteria identified in Fig. 1 for experimental design represent important components needed to understand the overall reliability for interpreting how the study was conducted. The criteria used to evaluate in vivo mammalian studies were selected by combining elements recommended in various sources, including the OECD guidelines [49–53] and criteria adopted and applied by both Fernández-Cruz et al. [9] and de Ruijter et al. [3]. Specifically, Fernández-Cruz et al. [9] identify the importance for reporting the species, total number of animals, number of animals per test group, details regarding the housing and feeding conditions, administration route, homogeneity of particles in the exposure media, particle stability and the frequency and duration of exposure. Specific guidance with respect to best practices for the various study design criteria can also be obtained from standard OECD test guidelines [49, 50, 53]. Additionally, de Ruijter et al. [3] include a criterion related to verifying the exposure, both in the exposure media and ideally data that can confirm and quantify the extent to which particles might be present in tissues observed to be adversely affected. Verification of actual exposure concentrations provide stronger interpretation of dose-response relationships, as compared to data limited to relying on nominal concentrations [17, 48, 54–56].

The criterion aligned to the reporting of particle concentration units for both in vitro and in vivo test systems has been included to capture and acknowledge attempts by different groups to report concentrations using more than one dose metric [57]. While mass-based dose metrics are typically used in chemical risk assessment, including additional dose metrics, such as particle count and/or particle surface area, can strengthen additional mechanistic insight between exposure and observed adverse effects. Furthermore, the use of negative and/or positive controls for both in vitro and in vivo effects studies represent opportunities to consider the relative performance of the study. For instance, reporting results in the absence of any known stressor (i.e., inclusion of a negative control) helps to characterize a baseline from which to compare results, whereas inclusion of a stressor known to trigger the adverse effect under investigation (i.e., positive control) can provide insight regarding how NMPs being evaluated compare against other known toxicants and can help to demonstrate that the test system is performing as expected. If possible, inclusion of another type of particle, either known to trigger a toxicological response or which might be considered toxicologically benign, could enable additional interpretation of test results that may provide additional mechanistic understanding and/or perspective with respect to environmentally relevant exposures.

Criteria suggested for evaluating in vitro effects studies used to assess human toxicity endpoints are generally similar to those identified for in vivo studies. Specifically, studies should report the cell model used and all the necessary information needed to evaluate how the in vitro system was sourced and maintained (e.g., submerged or cultured at air-liquid interface). Furthermore, and consistent with the evaluation of in vivo studies, the number of independent replicates per exposure dose, the administration route, the homogeneity of the particles themselves in the exposure media, their stability and the frequency and duration of exposure are also identified as important components to characterize and report.

### Applicability for risk assessment

The NMP-TSAT described here has been developed with the specific intention of evaluating effects studies for the purposes of screening and prioritizing for use in characterizing the potential human health risks of NMPs. Consequently, it is acknowledged that while not all effect studies will contain the necessary information for use in quantifying risk [17], studies that do not meet the criteria described under this category may still add value to our overall scientific and mechanistic understanding. The data reported in effect studies can be filtered in different ways, which can effectively enable the screening and prioritization of studies for addressing different research questions [40].

The adoption, application and reporting of statistical methods used to analyse data observed from an effects study can serve a variety of purposes. Statistical analysis can help to evaluate the relevance and reliability of various study design elements and is critically important when considering the interpretation of results against various endpoints measured and reported, including interpreting the level of confidence associated with the reporting of dose-response relationships. The concentration range and environmental relevance of the particles tested in effects studies can also be used in helping to evaluate various questions. For instance, effects studies that use very high concentrations, while not necessarily reflective of environmentally-relevant concentrations and therefore not necessarily beneficial for evaluating human health risk [18], may still provide insight into mechanisms of action or help provide insight for other researchers in selecting concentrations for future studies that may trigger certain endpoints. Similarly, studies that evaluate a single size, shape and polymer type may prove beneficial towards evaluating mechanistic relationships between those specific properties and an endpoint of interest but cannot currently be used to extrapolate to heterogenous mixtures of NMPs found in the environment [17].

The criteria used to evaluate applicability for human health risk assessment are those aligned to the endpoints reported in the study, the dose-response relationship, and the reporting of effect threshold values, such as the NOAEL, LOAEL or BMD. It is acknowledged that endpoints are typically reported at the sub-organismal level, such as cellular and molecular level biomarker effects [20]. While helping to elucidate mechanisms of action, including molecular initiating and key events along a potential AOP [58], additional information related to how these observations may adversely affect later key events and ultimately an adverse outcome (i.e. higher levels of biological organization) are needed to extrapolate to human health risk. Similarly, dose-response relationships based on adverse effects observed at higher levels of biological organization, and which include at least three concentration doses plus the control, and with a concentration range ≥ 3x, are considered adequate for the purposes of assessing risk. Ideally, the information presented in the dose-response would also provide a sufficient level of information to enable an effect threshold to be derived, with error data to characterize the 95% confidence interval, standard error or standard deviation. For studies that do not specifically report an effect threshold, evaluation of the data reported for the dose-response should be conducted to determine if a BMD can be extrapolated from the available information.

### Literature review

The studies identified and included for evaluation using the NMP-TSAT are based on a literature review using the PubMed search engine, provided by the National Center for Biotechnology Information, and using the keywords ‘microplastic’ AND ‘toxicity’. Studies reporting original research for both in vitro and in vivo-based effects were prioritized for evaluation. Additional sources of data include the identification of synthetic fibres from the reference list obtained from a UK Health and Safety Executive (HSE) [59] report summarizing the hazards and risks of fibres, which was further supplemented by a keyword search for ‘synthetic fibre’ AND (‘toxicity’ OR ‘health’) using PubMed up to March 2021, and a review of studies identified by Rahman et al. [18] as part of their scoping review related to studies reporting on the human health risks of NMPs. Furthermore, a review of studies identified in the development of health-based recommendations for NMPs in drinking water have also been included [40]. As a complement to the NMPs studies, and in order to provide a broader context and interpretation of the results reported for NMPs, an additional group of studies reporting on the effects associated with exposure to cellulose particles is also included in the evaluation. To this end, studies were also identified using the PubMed search engine using the keywords ‘cellulose’ AND ‘toxicity’, which was further supplemented by studies identified by Endes et al. [60] and Dourado et al. [61]. Original research reporting both in vitro and in vivo effects data were prioritized for evaluation. While every effort has been made to identify all relevant studies for assessing and evaluating their relative performance against various QA/QC elements identified above, we acknowledge that the approach taken to identify studies is not comprehensive and that some studies may have been missed based on limitations associated with how the literature search was performed. For instance, the keyword ‘microplastic’ has only been in use since about 2004 [62]. Consequently, studies reporting effects on NMPs prior to this date would not have included reference to these types of particles as part of their keywords. Nevertheless, an effort to include studies prior to 2004 that report adverse effects for NMPs have been identified by screening for relevant references cited in the various studies identified through our initial literature search.

### Study evaluations

Tables S1 and S1 (Supplementary Information) provide guidance in relation to scoring each of the criteria with respect to the information being adequate [2], adequate with restrictions [1], or inadequate (0) in regards to documentation. Consistent with the approach adopted in previous method evaluation papers [3, 27, 37, 39], and noted above, we emphasize that the scores assigned for each study should not be perceived as a judgement indicative of the relative value of the research, i.e., a paper scoring low on a certain criterion could still provide valuable information regarding other potential insights. Problem formulation is therefore an important element to understand, in that depending on the purpose of an effects study the results may or may not help to inform the decision-making process with respect to assessing risk. A weight-of-evidence may be assembled, for instance, regarding an effect mechanism, but the mechanism may not necessarily be relevant regarding human health implications. The primary objective of the evaluation criteria developed and applied in this study is thus aimed towards providing insight regarding QA/QC criteria that could be improved in future studies in order to better inform hazard characterization and the application of a quantitative risk assessment. Ideally, studies should score a value of ‘1’ against all criteria to demonstrate their relevance towards assessing risk to human health. However, based on the results of de Ruijter et al. [3], who observed that no ecotoxicological effects studies received a score of ‘1’ against all criteria, we have implemented a screening approach that adopts the use of a minimal set of critical or ‘red’ criteria, similar to the approach used by Fernández-Cruz et al. [9] and which have been agreed by participants of the Health Effects Workshop sponsored by the Southern California Coastal Water Research Project https://www.sccwrp.org/about/research-areas/additional-research-areas/trash-pollution/microplastics-health-effects-webinar-series/. Criteria highlighted in red in Fig. 1 have been identified as representing the recommended minimal level of information required for evaluating a study as fit-for-purpose, i.e., for human health risk assessment [9]. It is important to note that the ‘red’ criteria can be modified depending on the purpose for evaluating and prioritizing toxicity effects studies, such as through the toxicity tool [40]. All studies identified in the literature review were evaluated using the NMP-TSAT by at least two individuals, including contributing authors and those identified in the acknowledgements. Individuals contributing to the evaluation of studies enabled access to varying expertise and include representation from academia, government and industry. Through the evaluation and feedback acquired through the review and assessment of each of the studies by varying individuals, criteria and scoring guidance have been refined.

## Results and discussion

### Literature review

A total of 76 studies reporting data from both in vivo and in vitro effect test systems representing either inhalation or oral ingestion exposure pathways were identified and evaluated. With the aim of providing a broader context and interpretation of the results reported for NMPs, 16/74 studies include data reported for cellulosic materials. Given the current understanding that NMPs represent a heterogeneous mixture of plastic particles of varying polymeric composition, size and shape, the inclusion of effect studies performed on natural cellulosic polymers provides an opportunity to compare and contrast with respect to QA/QC practices but can potentially also provide insight regarding similarities and differences with respect to toxicological mechanisms of action. Studies include 24 oral ingestion and 16 inhalation exposure in vivo effect studies on NMPs, and 11 gastrointestinal models and 9 respiratory models from in vitro-based NMP studies. A summary of the QA/QC evaluation of these studies is presented below, subdivided into the three main aspects of NMP-TSAT: particle characterization, study design and applicability of risk assessment.

### Particle characterization

The sizes of particles tested are generally similar for both in vivo and in vitro effect studies, with the median particle size tested observed to be 2.2 μm and 0.5 μm, for in vivo and in vitro studies respectively, whereas the respective mean particle sizes are 12.2 ± 31.7 μm and 15.2 ± 43.3. The majority of studies tested particles < 5 μm, with a minimal particle size of 10 nm and a maximum particle size of about 200 μm. This is in contrast to ecotoxicity studies, where de Ruijter et al. [3] observed that only about 30% of studies tested particles < 10 μm. Consistent with the observations of de Ruijter et al. [3] the majority of studies evaluated using the NMP-TSAT have been performed on spheres (≈60%), consisting primarily of polystyrene (≈46%). The other plastic polymers tested include a small number of studies that report results for polyethylene (10%), polyvinyl chloride (PVC) (5%), polypropylene (3%) and polyethylene terephthalate (PET) (3%). A limited number of other types of plastic polymers have been investigated from in vivo and in vitro effect studies, with nylon, polyurethane and acrylic-ester being tested in a small number of in vivo effect studies [63–66], generally representative of inhalation exposure.

A curious observation from the evaluation of studies relates to the source of particles that researchers identify. Major sources of NMPs can be attributed to five companies who are identified as providing particles for approximately 45% of studies. The companies are BaseLine Chromtech Research Centre (China), Sigma-Aldrich (USA), Cospheric (USA), Kisker Biotech (Germany) and Microspheres-Nanospheres (USA). Information obtained from the product data sheets for the particles used in the studies suggests that the particles have been produced for purposes other than for use in testing potential human health effects of environmental NMP exposure. For instance, some particles are described by suppliers as being monodisperse for the purposes of use in immunodiagnostic assays as size standards for calibrating analytical equipment or as substrates or supports for immunologically based reactions, tests and assays. Additionally, particles may be used to support cellular biology applications, typically by providing a substrate for binding protein ligands. In some instances particles can be obtained in powder form, but in most cases, the particles are obtained as a liquid suspension. BaseLine Chromtech Research Centre, for example, supply their polystyrene particles in a 1:1 ethanol/water solution, with other suppliers describing the particles as being dispersed in an aqueous solution. Although data obtained from the product data sheets might be used to evaluate the particle size distribution for the particles, details related to the purity of the particles themselves are less well understood. Of particular concern is the lack of information reported on the levels of unreacted monomer or impurities that may be present in the polymer, whereby residual levels of styrene in polystyrene may represent a potential chemical contaminant that may influence toxicity test results [67]. The extent of surfactants, antimicrobials and/or dispersants present in the product, or which may have been used during their manufacture, is also typically unknown. The observation that some particles are supplied in an ethanol/water solution is illustrative of a scenario whereby the particle product matrix or residual chemicals, such as ethanol, surfactants and/or dispersants may potentially confound interpretation of observed adverse effects [68–70]. Finally, no information is available regarding how the particles themselves were produced at the above noted companies, and it is unclear if it is appropriate to extrapolate the results of these toxicity studies to inform a human health risk assessment of NMPs encountered in the environment.

**Fig. 2:**
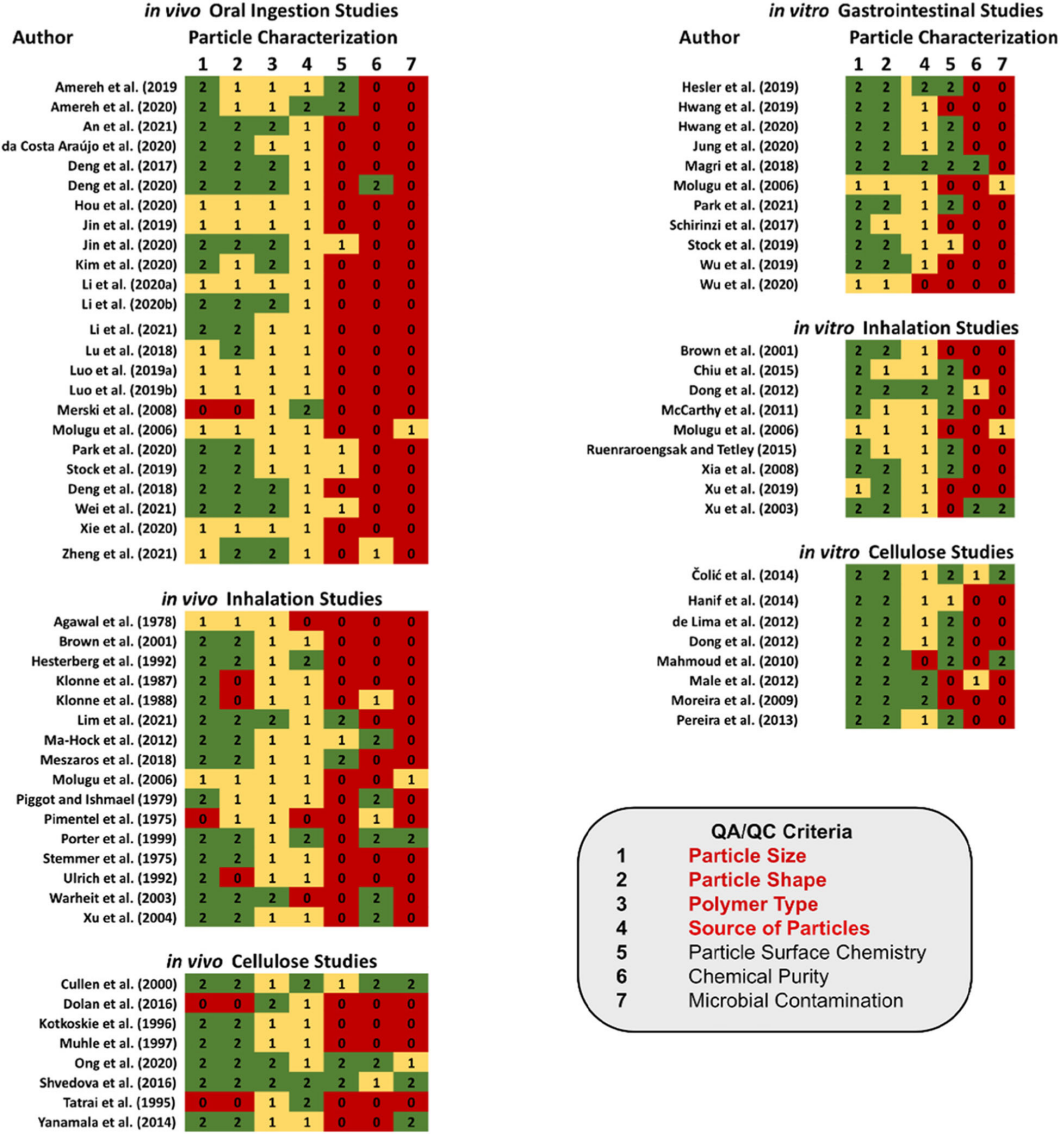
Summary of evaluation scores for QA/QC criteria aligned with the reporting of particle characterization components of studies assessed by the NMP-TSAT. Please note that some studies report results for both in vitro and in vivo toxicity effects, such as Brown et al. [82], Molugu et al. [72], and Stock et al. [83]

Figure 2 summarizes the individual scores from all studies for criteria under the particle characterization category. A general observation is that all studies generally report details as adequate [2] or adequate with restrictions [1] aligned to each of the red criteria, i.e. particle size, shape, polymer type and the source of the particles. Conversely, very few studies report any details regarding the particle surface chemistry, the chemical purity or test for the presence of microbial contamination, such as endotoxin. It is observed that no studies related to an oral ingestion exposure pathway for either in vivo or in vitro included adequate testing for endotoxin, with only one in vitro and one in vivo study reporting on chemical contaminants that may potentially be present in the particles tested [71]. Testing for endotoxin and other chemical contaminants, on the other hand, appear to be slightly better addressed for inhalation exposure scenarios and for studies evaluating the adverse effects of cellulose particles [65, 72–75]. Four studies (3 in vivo, 1 in vitro) received a non-zero score against all QA/QC criteria aligned with the characterization and reporting on cellulose particles [73–76], whereas none of the studies reporting on NMPs received all non-zero scores. Biofilm and pathogenic organisms have been reported to colonize hydrophobic nonpolar surfaces, characteristic of NMPs, faster than hydrophilic surfaces, which has resulted in an increased interest in relation to the role that NMPs may play as vectors for pathogens [77–80]. The microbial inhabitants associated with MPs include potentially pathogenic bacterial species, like *Pseudomonas* spp., *Vibrio* spp., *Campylobacter* spp. and *Escherichia coli* [81]. Given the potential that NMPs can become contaminated by various microorganisms, which could trigger a toxicological response in the model test system, studies that characterize and quantify the presence or absence of an endotoxin that may have contaminated the test material prior to administering the dose would therefore prove beneficial towards demonstrating correlations between the particles themselves and an observed adverse effect.

Reporting the surface chemistry of particles is most routinely included in in vitro effects studies [20, 50, 69, 83–93]. This observation is consistent with the objectives of many in vitro studies, which attempt to evaluate how varying properties of particles, such as size, shape and surface charge, influence the interaction between particles and cells used in various bioassay test systems. A good example is the study of Magri et al. [89], who attempted to generate environmentally relevant nano-size PET particles using a laser ablation method. In their study, Magri et al. [89] perform a number of tests aimed at characterizing and quantifying the physicochemical properties of the particles generated. An important aspect they consider relates to the surface charge of the particles, which they describe as a negatively charged surface, and is confirmed by the presence of carboxylic acid groups on the surface of the particles and zeta-potential measurements. The authors also consider the potential influence that the negative charge has on the stability of the particles within the in vitro assay test medium, as well as the potential influence of the formation of a protein corona on particle surfaces [89]. The added value regarding the quality of information presented by Magri et al. [89] thus provides opportunities for enhanced mechanistic understanding of the behaviour and fate of the particles in the test system and strengthens the interpretation and confidence of study results by reducing potential sources of uncertainty regarding QA/QC components of the study.

### Study design

Several different types of in vivo mammalian test models and endpoints have been used in testing the adverse effects of both NMPs and cellulose particles associated with both inhalation and oral ingestion exposure pathways. While the use of rodents represent the majority of studies, there are a few studies using other animal models, including the use of rabbits [72], pigs [94] and zebrafish [95]. The endpoints investigated include mortality, behavioural changes, reproductive effects, adverse effects on gut microbiota, metabolism and adverse effects on various internal organs (e.g., liver, lung, heart, spleen, reproductive tissues), including effects on organ and body weight and inflammatory biomarkers.

Similarly, in vitro effect studies target a variety of cell bioassay test systems and endpoints related to both inhalation and oral exposure pathways, with cell viability/cytotoxicity, inflammation, and cellular uptake generally dominating. Consequently, due to the variability associated with the test systems that have been applied, we emphasize that the aim of the evaluation of QA/QC criteria aligned with study design is to primarily assess the reporting of fundamental test system components. The evaluation can therefore provide a quantitative approach towards a Tier 1 screening and prioritization level of assessment, aimed at prioritizing studies that are found to address and report QA/QC criteria to a satisfactory level of completeness. The evaluation criteria, however, do not provide an indication of the overall quality of the interpretation of results obtained from the study, which would require Tier 2 expert elicitation from individuals with specific expertise and understanding related to the test system used and how to best interpret data obtained with respect to the endpoint measured.

**Fig. 3:**
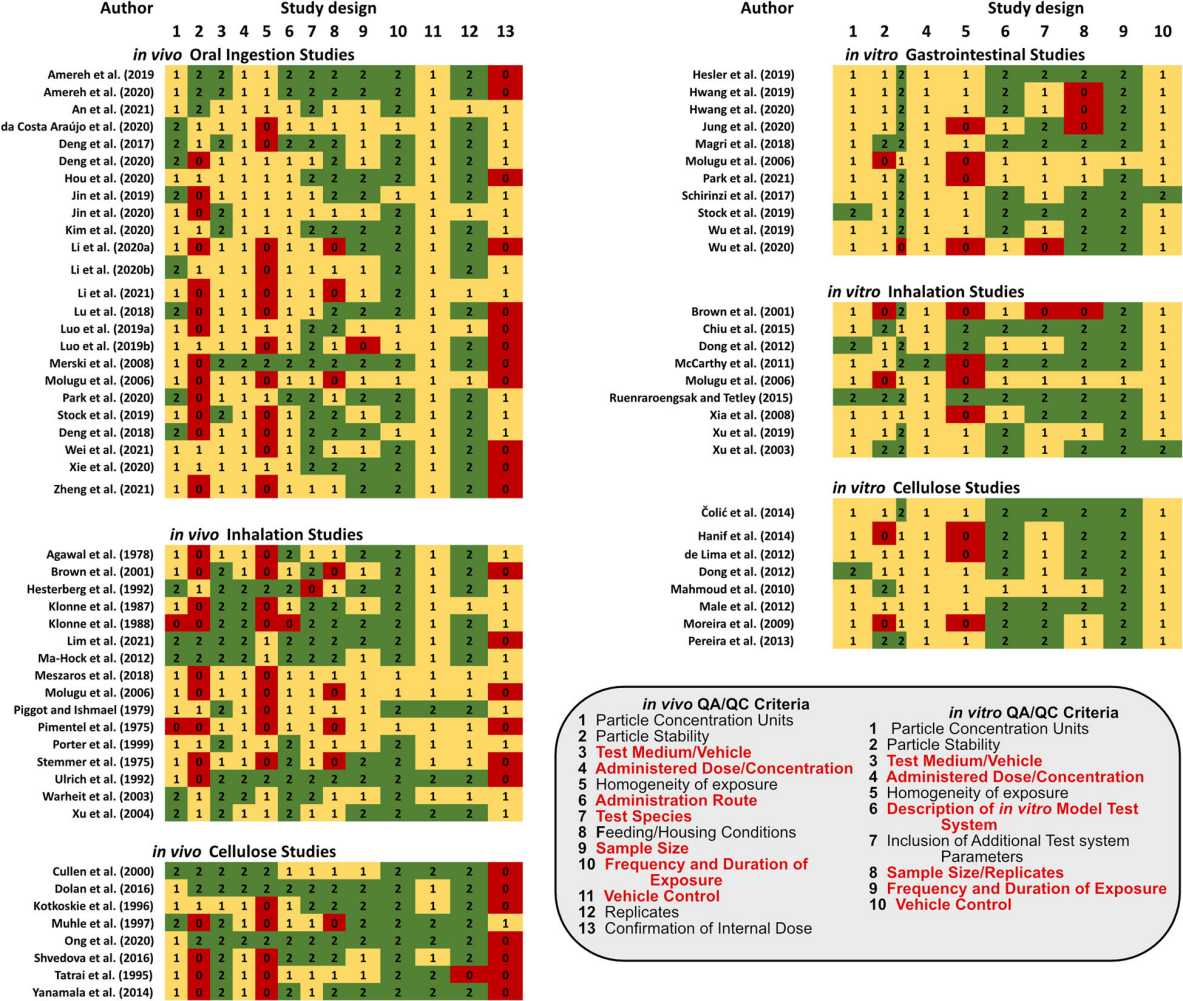
Summary of evaluation scores for QA/QC criteria aligned with the reporting of study design components of studies assessed by the NMP-TSAT. Please note that some studies report results for both in vitro and in vivo toxicity effects, such as Brown et al. [82], Molugu et al. [72], and Stock et al. [83]

Figure 3 summarizes the evaluation scores for each of the study design QA/QC criteria for both in vivo and in vitro studies. In contrast to the reporting of particle characteristics, there are several studies that receive non-zero scores against all QA/QC criteria aligned with study design for NMPs [65–67, 83–85, 92, 95–101]. The adequate use and reporting of study elements, such as the use of controls and the reporting of frequency and duration of the study are observed to be satisfactorily reported by all studies. Similarly, studies tend to provide the minimally sufficient level of information reporting on the particle concentration, the test medium or vehicle used to deliver a dose, the administration route, the test species or bioassay details and the size of the samples and numbers of replicates. Areas where studies have not performed as well relate to characterizing and quantifying the stability and homogeneity of the particles in the exposure medium, and specific to in vivo studies, confirmation of internal dose.

In their study investigating the inhalation exposure of NMPs (100 nm polystyrene beads), Lim et al. [102] adopt a modified OECD 412 test guideline (28-day (subacute) inhalation toxicity study [53]) to evaluate effects using a whole-body inhalation exposure system. The study includes detailed information related to the exposure test chamber, including quantification of particles in the test chamber to which the animals were exposed, exposure concentrations reported as both mass/volume and count/volume basis, detailed descriptions of the feeding and housing conditions of the animals, the species and sex, and the inclusion of the OECD-recommended numbers of animals per test group [53]. However, while Lim et al. [102] include histological analysis of tissue samples, the data are insufficient to confirm the internal dose of particles. In many instances a score of ‘1’ has been assigned in relation to confirming the internal dose based on semi-quantitative estimates or qualitative information obtained from histological images, such as from Ma-Hock et al. [97], who also score relatively well against all QA/QC criteria for their short-term inhalation study on acrylic-ester particles (< 1.5 μm).

In their study investigating the influence of surface charge in relation to adverse effects on primary human alveolar macrophages (MAC), primary human alveolar type 2 (AT2) epithelial cells and a unique human alveolar type 1 epithelial cells (TT1), Ruenraroengsak and Tetley [92], include information on the stability of the particles in the test system with concentrations reported on both a mass/volume and mass/area basis. The study also includes the use of both vehicle controls and positive controls, which were used in evaluating the performance of the lactate dehydrogenase assay (LDH) [87]. Providing a relatively high-quality level of QA/QC information with respect to the study design, the observation that all types of particles tested were internalized by TT1 and MAC cells, whereas only a small fraction of positively charged particles were internalized by AT2 cells, strengthens confidence in the overall interpretation of data presented. As noted above, improved handling and reporting of QA/QC criteria greatly helps in reducing potential uncertainties that may arise from other studies where information presented may potentially be ambiguous and/or simply not reported.

Raising awareness on how the lack of reporting and/or demonstrating steps taken to comply with QA/QC is particularly important when authors imply human health implications or attempt to position their results within the context of human health risk assessment. Studies investigating the human health risks of cellulosic materials, for instance, provide potential opportunities for learnings that could be applied in future studies attempting to characterize and quantify the human health risks of NMPs. The study of Ong et al. [74], who report on the adverse effects associated with dietary exposure to fibrillated cellulose in Sprague-Dawley rats using the OECD-408 90-day dietary test guideline [49], may provide an example upon which to consider. As shown in Fig. 3, Ong et al. [74] receive a score of ‘2’ against 11/13 criteria. Given that the guidance used for the scoring criteria are partly based on OECD guidelines, this result is not too surprising. However, since the authors conducted their study following OECD guidance, the NOAEL reported (2194.2 mg/kg/d (males); 2666.6 mg/kg/d (females)) [74], lends itself well for direct use within a regulatory context. A good example to compare against is the study of Merski et al. [103], who apply the OECD guidelines to their study of repeat oral ingestion of a spunbound, nonwoven polymer fabric consisting of polyethylene and polyethylene terephthalate microparticles and found no apparent toxicity at dietary levels up to 5%. While the various study design criteria were reported to a sufficient level of detail and can thus provide a useful line of evidence, the details reported in relation to the particle characteristics (Fig. 2) are poorly documented, resulting in uncertainty in applying the study results for assessing the potential risk of NMPs.

### Applicability for risk assessment

**Fig. 4:**
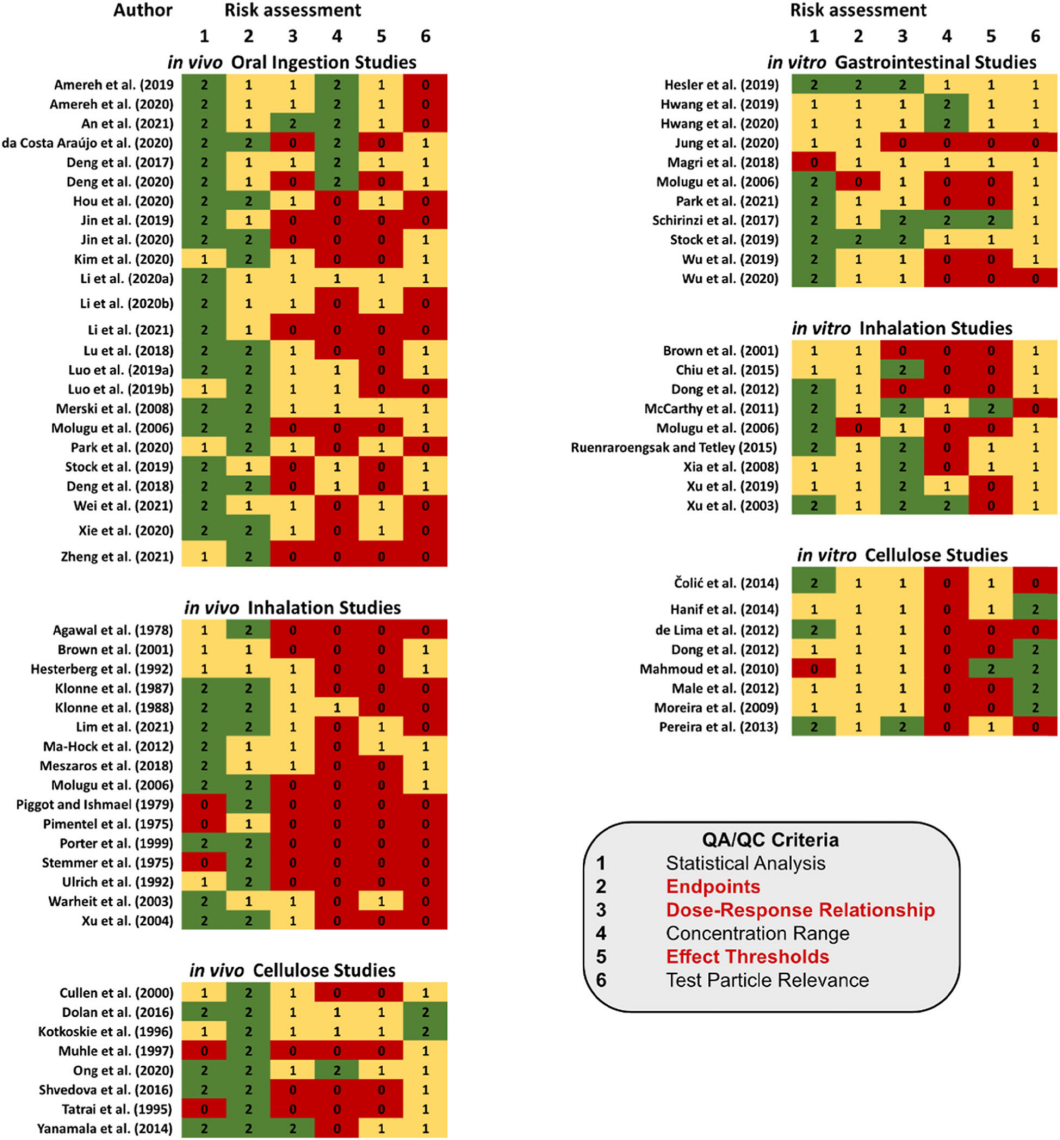
Summary of evaluation scores for QA/QC criteria aligned with the reporting of data for use in assessing human health risks components of studies assessed by the NMP-TSAT. Please note that some studies report results for both in vitro and in vivo toxicity effects, such as Brown et al. [82], Molugu et al. [72], and Stock et al. [83]

A major objective behind the development and application of the NMP-TSAT is in helping to identify studies for use in evaluating human health risk, and to inform the derivation of regulatory and non-regulatory values for microplastic in exposure media such as drinking water. The results obtained from the NMP-TSAT with respect to criteria aligned with applicability for risk assessment are shown in Fig. 4. A general observation is that while studies provide an adequate level of information related to the statistical methods used in analysing the endpoints measured, few studies provide information that support that the particles tested are representative of NMPs found in the environment, or that the concentrations tested are representative of environmentally relevant exposure scenarios. Furthermore, approximately half of all in vivo-based studies are based on using ≤2 concentration exposures, which represents an insufficient level of information to derive an effect threshold value. Nevertheless, two in vivo studies reporting effects of NMPs are identified as receiving non-zero scores against all criteria related to the application of data for use in risk assessment. Li et al. [104] report on the effects that 10–150 μm polyethylene particles have on the distribution of gut microbiota and inflammation. The mass used ranged from 6 to 600 μg for a single type of particle and shape, but which represents an environmentally relevant particle size distribution. Although the authors do not report either a NOAEL or LOAEL, data presented suggest the potential to extrapolate a LOAEL in relation to the highest exposure concentration [104]. Merski et al. [103], on the other hand, report on the oral toxicity and mutagenicity of spun-bound polyethylene and PET polymers derived from fabric materials. The concentration range presented, however, is based on a weight percentage of particles in food, representing 0.5, 2.5 and 5%, which is assumed to have an environmental relevance [103]. Additional relevance towards NMPs present in the environment can be perceived based on the use of two different types of polymeric materials. Similar to Li et al. [104], the authors do not report an effect threshold value [103], although data presented potentially imply the NOAEL to be aligned with the highest exposure dose.

While five in vitro-based studies received non-zero scores against all risk assessment criteria [83, 86, 87, 99, 105], a major challenge relates to the lack of tools currently available to extrapolate in vitro results to in vivo scenarios [20, 26, 106]. Thus an important research need in helping to strengthen the use of in vitro data in regulatory risk assessment pertains to the development of quantitative in vitro-to-in vivo extrapolation (QIVIVE) models [35, 107–111].

When considering studies reporting effects for cellulose particles, three studies receive non-zero scores against all criteria [74, 112, 113]. Dolan et al. [112] investigated the effects of adding pecan shell fibre to food based on results obtained from a repeat dose 90-day oral toxicity study in rodents (OECD 408) [49]. The concentration is estimated to range from between 3571 to 10,714 mg/kg/d (5–15% of diet), with no effect on body weight or on any toxicologically relevant endpoints [112]. A NOAEL of 9947.5 mg/kg/d (males) and 11,082.8 mg/kg/d in rats related to dietary exposure is reported. In their study related to the adverse effects of microcrystalline cellulose, Kotkoskie et al. [113] dosed Sprague-Dawley rats with exposures of between 500 and 5000 mg/kg/day in tap water by oral gavage. Following the OECD 408 [49] repeat dose 90-day oral toxicity study recommendations no toxicologically significant effects or lesions were observed, resulting in a NOAEL of 5000 mg/kg/d in rats being reported [113]. Finally, Ong et al. [74], who also report results obtained from the OECD 408 [49] 90-day oral toxicity test, report a NOAEL for fibrillated cellulose in the diet of Sprague-Dawley rats of 2194.2 mg/kg/d (males) and 2666.6 mg/kg/d (females) based on the analysis of several endpoints, including body weight, food consumption, ophthalmologic evaluations, hematology, serum chemistry, urinalysis, post-mortem anatomic pathology and histopathology.

It is proposed that the results obtained from the cellulose particle studies may provide a benchmark upon which to compare effect threshold results obtained from NMP studies of similar quality. Unfortunately, the studies identified above receiving non-zero scores with respect to risk assessment criteria for in vivo oral exposure to NMPs, cannot currently be directly compared to the cellulose particle studies due to various differences in study design and units reported. The LOAEL estimated from Li et al. [104], for instance, is 600 μg/d, estimated to have been consumed in 3 g of diet each day by mice, representing approximately 0.02% of dietary exposure, whereas an estimated NOAEL based on results reported from Merski et al. [103] may be representative of an exposure of NMPs of 5% from dietary exposure. Considering that the exposure doses used in the NMPs studies are lower than those used in the cellulose particle studies, the lack of effects at the higher cellulose exposure concentrations may represent a benchmark threshold for comparing NMP study results, although caution is warranted not to overinterpret these results, such as through the application of read-across. Such an approach, however, may provide opportunities to contextualize how results obtained from NMP effect studies relate to exposures to other non-digestible particles found in typical human diets.

### Evaluation scores

As discussed and illustrated above, the NMP-TSAT can be used to evaluate the reliability and relevance of any in vivo or in vitro NMP toxicity study based on assessing how studies perform against a suite of generic criteria, including:

- Test substance identification,
- Test system characterization, and.
- Study design description.

For both in vivo and in vitro studies, criteria must be assessed against the adequacy of information presented, ranging from adequate (score = 2), adequate with restrictions (score = 1) or inadequate (score = 0). Because scores of between 0 and 2 can be assigned to each criteria, one approach towards screening and prioritizing studies may be to assume that the sum of the scores can be computed and the relative reliability of the study evaluated based on the overall score, particularly for studies where all criteria receive a non-zero score. The total assessment score (TAS) possible for in vivo studies is 52, whereas for in vitro studies it is 46, which is based on receiving a maximum score of 2 against 26 and 23 criteria, respectively. The maximum TAS obtained for an in vivo study evaluated here was 44 (85%), assigned to the cellulose particle study of Ong et al. [74]. In comparison, the maximum TAS score obtained for an in vivo NMP study was 37 (71%), assigned to the inhalation in vivo studies reported by Lim et al. [102] and Ma-Hock et al. [97]. The maximum TAS for in vitro studies was 34 (74%), assigned to the studies of Hesler et al. [86] and Xu et al. [67]. Minimum TAS of 12 (23%) and 16 (35%) were assigned to in vivo and in vitro studies respectively, and the mean TAS were 28 (53%) and 26 (56%), respectively. While this may appear to be an intuitive approach, care should be taken not to overinterpret the comparison of overall scores between studies.

The scoring approach used by Fernández-Cruz et al. [9] screens and prioritizes the reliability of studies based on combining information regarding how they score overall and in relation to specific critical criteria. In that approach high quality studies, identified as those that have > 85% of the questions receiving the maximum score, and those that meet all critical criteria would be prioritized [9]. In instances where 61–85% of the questions receive the maximum score, and all critical criteria are met, studies are deemed to be reliable with restrictions [9]. For studies having < 61% of questions receiving the maximum score or if any of the critical questions are not addressed the study is deemed to be unreliable [9]. When considering the results obtained from the NMP-TSAT using the approach of Fernández-Cruz et al. [9], only the study of Ong et al. [74] reporting effects of cellulose particles would be prioritized as reliable, and no NMP studies would be deemed reliable. Seven studies reporting the in vivo effects of NMPs would be identified as reliable with restrictions [66, 97, 98, 102, 114–116], whereas nine in vitro effect studies would fall into this category [67, 83, 85, 86, 89, 90, 92, 99, 101]. Consequently, > 80% of studies are identified as not reliable using the prioritization approach described by Fernandez-Cruz et al. [9] and the NMP-TSAT criteria.

In their prioritization approach, de Ruijter et al. [3] suggest that all criteria are equally important, therefore only studies receiving a score of ‘1’ against all criteria can be considered adequate. We note, however, that if a study receives a score of ‘1’ against all criteria, the TAS would represent 50% of the maximum score possible, which when combined with the cut-off values defined by Fernández-Cruz et al. [9] would result in the study being defined as unreliable. This is because Fernández-Cruz et al. [9] use cut-off values based on scoring each criterion on a value of ‘0’ or ‘1’, whereas de Ruijter et al. [3] introduce an additional score of ‘2’, enabling studies to be differentiated based on providing a higher level of information. Thus, the use of cut-off values using the scoring system of de Ruijter et al. [3] would require modification to enable studies to be screened and prioritized based solely on their TAS. In both the study of de Ruijter et al. [3] and in the data reported here, none of the studies receive non-zero scores against all criteria, thus none of the studies can be defined as ‘adequate’ using the approach suggested by de Ruijter et al. [3].

Given the challenges associated with prioritizing studies based on ranking them according to their TAS we suggest the use of an alternative, more flexible approach. Specifically, we suggest that studies initially be screened on how they score against each of the critical red criteria, highlighted in Fig. 1. Studies that score a minimum of ‘1’ against each of the red criteria, regardless of how they rank according to their TAS, would be prioritized as part of a Tier 1 screening and prioritization phase.

Using this approach we identify 10 oral ingestion [96, 104, 114–121] and 2 inhalation in vivo studies [97, 102] that score at least 1 against all red criteria (Table 1). Six of those studies receive a TAS < 60%, but since they report a sufficient level of detail aligned to each of the critical QA/QC criteria, their results may be useful for human health risk assessment. For instance, study results include a dose-response relationship based on > 3 concentration doses, thus an evaluation of these studies at a higher tier of assessment may potentially prove beneficial. Since the NMP-TSAT is not sufficient to evaluate the validity and quality of the interpretation of study results, experts with the necessary level of expertise to assess the reliability of toxicological endpoints should be consulted.

**Table 1:** Summary of total assessment scores (TAS) and QA/QC criteria strengths and weaknesses of in vivo studies prioritized based on adequate evaluation of critical QA/QC identified as part of a Tier 1 screening assessment. Strengths and weaknesses listed reflect how the data support or limit, respectively, the interpretation of study results within a risk assessment context. Strengths and weaknesses are thus context-specific, and those listed as strengths here may be perceived as weaknesses under another context, and vice versa. Additional study information included in the Supplementary Information

Author	Strengths	Weaknesses	TAS (/52)
Lim et al. (2021) [102]	Excellent reporting of study design characteristics; inclusion of both sub-organismal and organism level endpoints reported helping to inform potential mechanisms of toxicological action	Monodisperse polystyrene spherical particles – unclear how to extrapolate results to environmentally relevant exposures.	37
Ma-Hock et al. (2012) [97]	Excellent reporting of particle characteristics and exposure conditions, including an estimate relating external exposure to an internal dose; NOAEL reported based on no adverse effects up to the maximum concentration tested.	Single type of polymer tested at only two concentration doses, plus control – acrylic ester copolymer – unclear how to extrapolate results to environmentally relevant exposures.	37
Ong et al. (2020) [74]	Study conducted according to repeat oral dose OECD 408 guideline with excellent reporting for all three areas evaluated, including particle characteristics, study design and application for risk assessment – NOAEL reported.	Study targets the evaluation of adverse effects related to repeated dietary ingestion of fibrillated cellulose – unclear how to read-across to NMPs.	44
Amereh et al. (2019) [115]	Good characterization of particles, which include two different sizes in the sub-micron range; particles tested at both high and environmentally relevant concentrations, LOAEL can be extrapolated.	Single type of polymer tested – polystyrene spheres; particles dosed into drinking water, unclear actual delivery and/or homogeneity of exposure; adverse effects limited to sub-organismal level endpoints only.	34
Amereh et al. (2020) [114]	Good characterization of particles, which include two different sizes in the sub-micron range; particles tested at both high and environmentally relevant concentrations, LOAEL can be extrapolated.	Single type of polymer tested – polystyrene spheres; particles dosed into drinking water, unclear actual delivery and/or homogeneity of exposure; adverse effects limited to sub-organismal level endpoints only.	34
Deng et al. (2017) [116]	Inclusion of both sub-organismal and organism level endpoints reported helping to inform potential mechanisms of toxicological action, with exposure concentrations reported to represent environmentally relevant concentrations.	Two sizes of monodisperse polystyrene spheres; particles dosed into drinking water, unclear actual delivery and/or homogeneity of exposure; particles supplied as a dispersion in a solution containing 1:1 ethanol:water, unclear residual levels of ethanol in test system. Several comments published in the peer review literature raising concerns related to histopathological analysis and toxicokinetics.	34
Dolan et al. (2016) [112]	Study conducted according to repeat oral dose OECD 408 guideline with good reporting for study design and application for risk assessment – NOAEL reported.	Poor reporting of particle characteristics, where study reports adverse effects related to repeat dietary ingestion of pecan shell fiber, ground from pecan shells – unclear how to read-across to NMPs.	34
An et al. (2021) [96]	Lowest test concentration selected as being representative of concentrations reported for freshwater systems.	Monodisperse polystyrene spherical particles – unclear how to extrapolate results to environmentally relevant exposure; particles dosed into drinking water, unclear actual delivery and/or homogeneity of exposure; particles supplied as a dispersion in a solution containing 1:1 ethanol:water, unclear residual levels of ethanol in test system.	31
Kotkoskie et al. (1996) [113]	Concentration test range selected to be representative of concentrations in food product for human consumption, NOAEL reported.	Poor reporting of particle characteristics, where study reports adverse effects related to repeat dietary ingestion of cellulose fibers – unclear how to read-across to NMPs.	30
Park et al. (2020) [118]	Various organism and sub-organism level effects reported, including body weight, pathological effects in stomach epithelial cells, effects on reproduction and immune system, such as via Immunoglobins (Ig, IgA, total IgG, IgE, and IgM)	Monodisperse polyethylene particles – unclear how to extrapolate results to environmentally relevant exposure; particles dosed into drinking water, unclear actual delivery and/or homogeneity of exposure; unclear relevance of exposure concentration range used 3.75–60 mg/kg bw; questions related to the use of *p* < 0.5 as a measure of statistical significance.	30
Hou et al. (2020) [117]	Body weight and changes in organ coefficients, sperm damage analysis, including count, malformation, etc., inflammatory responses and apoptosis-related proteins and cells. A combination of both organism and sub-organism level endpoints. Concentrations of 0.1, 1, and 10 mg/L used in drinking water, with an estimate that mice drank 6–7 mL/d, resulting in a nominal derived concentration of 0.6–60 μg/d.	Poor particle characterization, with particle description limited to monodisperse polystyrene spherical particles – unclear how to extrapolate results to environmentally relevant exposure.	27
Li et al. (2020) [121]	Verification of particle size, shape and composition, while sufficiently reporting information for each of the critical criteria.	Monodisperse polystyrene spherical particles – unclear how to extrapolate results to environmentally relevant exposure; particles dosed into drinking water, unclear actual delivery and/or homogeneity of exposure; particles supplied as a dispersion in a solution containing 1:1 ethanol:water, unclear residual levels of ethanol in test system. Concentrations of > 7.18 × 10^9^ particles/L are perceived to be significantly greater than typical human exposure.	27
Wei et al. (2021) [119]	Verification of particle size, shape and composition, while sufficiently reporting information for each of the critical criteria.	Monodisperse polystyrene spherical particles – unclear how to extrapolate results to environmentally relevant exposure; particles dosed into drinking water, unclear actual delivery and/or homogeneity of exposure; particles supplied as a dispersion in a solution containing 1:1 ethanol:water, unclear residual levels of ethanol in test system.	27
Xie et al. (2020) [120]	Sufficient level of information reported for each of the critical criteria.	Monodisperse polystyrene spherical particles – unclear how to extrapolate results to environmentally relevant exposure.	27
Li et al. (2020) [104]	Sufficient level of information reported for each of the critical criteria.	Monodisperse polystyrene spherical particles – unclear how to extrapolate results to environmentally relevant exposure.	23

Table 1 provides a qualitative summary of strengths and weaknesses of the studies evaluated here using the NMP-TSAT. The strengths and weaknesses identified are limited to various QA/QC criteria, with strengths being identified for several studies in relation to particle characterization, adherence to OECD guidelines and the reporting of both sub-organismal and organism-level effects for potential use in human health risk assessment. Weaknesses are typically characterized by poor reporting and verification of test particle characteristics, with majority of studies testing monodisperse polystyrene spheres, resulting in a general lack of understanding of how these test particles might be used to extrapolate to the potential effects of NMPs encountered under environmentally relevant conditions. It should be noted that the strengths and weaknesses identified in Table 1 pertain to the purpose of studies for use in risk assessment. Depending on how the data are used, however, a strength identified here may represent a weakness in another context, and vice versa. For instance, where the purpose of the toxicity study is to investigate mechanistic relationships between specific properties of a particle (e.g. different polymer types, sizes, shapes, surface chemistry) and a toxicological effect endpoint, the use of monodisperse particles can be seen as an important strength. Alternatively, in instances where studies attempt to already extrapolate results using monodisperse particles to enable read-across to a heterogenous mixture of particles, such as is the case for many of the studies evaluated here, the challenges should not be underestimated, and in these instances the use of monodisperse particles represents a significant weakness. Additionally, there is a general reliance on nominal concentrations with several studies adding particles to drinking water with limited attempts to quantify or verify the actual exposure dose concentration; in these cases concentrations tested are most likely significantly above environmentally relevant concentrations.

Given that the evaluation performed using the NMP-TSAT is limited to assessing QA/QC criteria, and that the approach aims to prioritize a conservatively large number of studies, which includes studies that potentially provide an inadequate level of information against several criteria identified as less critical, it is important that all 15 studies in Table 1 be subject to a Tier 2 expert elicitation process. This is particularly important when identifying studies that might be used in helping to inform the decision-making process in relation to human health implications of exposure to NMPs, as the studies themselves adopt novel and non-standard approaches towards assessing a wide range of endpoints. Furthermore, the studies listed in Table 1 include a variety of endpoints, for which toxicologists representing different areas of expertise would be needed to provide a more in-depth evaluation of the study design and reliability of the data reported. We propose that by combining the screening and prioritization approach based on the application of the NMP-TSAT (i.e., Tier 1 evaluation) with expert evaluation for priority studies (i.e., Tier 2 evaluation), the method described here provides a standard evaluation and scoring procedure that will enable a transparent approach for assessing the relative quality and reliability of any in vivo or in vitro NMP toxicity study. The suggestion is that Tier 1 evaluation aims at examining the QA/QC reliability of studies, but not necessarily their relevance for use in risk assessment. Given the various toxicological endpoints reported from toxicity studies there is a need to receive input from multiple relevant experts, who can provide input regarding the relevance and reliability of the endpoints reported. Information obtained through from both levels of assessment should be transparently documented, with the use of NMP-TSAT providing an instrument for the Tier 1 evaluation. Coffin et al. [38], report results obtained from a Tier 2 evaluation, which complement the information presented here.

In principle, the results of a Tier 1 evaluation can be used to screen and prioritize studies, as well as help to inform on the quality and reliability of a given study for use in assessing potential health risks. Given the various potential technical limitations and challenges currently associated with testing the toxicity of particles, particularly NMPs, in both in vivo and in vitro test systems, their evaluation should be perceived as providing both a relative indication of the quality of studies currently available, as well as a tool for helping to identify and prioritize data needs in relation to considering critical assessment factors needed to inform the implications of NMPs on human health – useful for designing and informing future human health hazard characterization and risk assessment.

As discussed above, there is a need to develop QIVIVE tools for enabling results obtained from in vitro studies to be robustly used within a risk assessment context, which is currently unavailable for NMPs. Thus, prioritization of in vitro studies for the purposes of informing a human health threshold value would not be appropriate, with the results reported here potentially proving useful at a later date when reliable QIVIVE tools are available for NMPs. Nevertheless, the information obtained from in vitro studies may prove insightful towards improved understanding of mechanisms of action. As discussed by Jeong and Choi [122], results from a suite of in vitro assays can be evaluated to define relevant key events, such as reactive oxygen species (ROS) formation (KE1278). Indeed, the evaluation of information reported for all in vitro studies does provide support related to the importance of ROS formation as a potentially important key event for NMPs, potentially resulting in inflammatory responses or possibly vice versa, i.e. inflammatory responses triggering ROS formation. Studies aiding in the mechanistic understanding that can better define the relationship between the properties of NMPs and their potential to trigger ROS formation would prove beneficial. Of particular interest is the application of testing environmentally relevant NMPs. Studies have shown that weathering, for instance, can increase the ROS generation potential of NMPs, which may be mediated due to competitive processes that weathering also introduces, such as higher binding affinity of weathered NMPs to serum protein – a powerful ROS scavenger, which has been observed in in vitro culture medium [123].

## Implications

Given the exponential increase in the number of published NMP toxicity studies, there is a need for screening and prioritization tools, such as the NMP-TSAT presented here, aimed at enabling a transparent and consistent approach for evaluating their relevance and reliability for human health risk assessment. When combined with data filtering tools [40], a tiered approach can help to prioritize resources towards the evaluation of studies in the context of human health risk assessment. A tiered approach that initially screens and prioritizes against critical QA/QC criteria, will help to ensure that high quality, fit-for-purpose studies are used for human health risk assessment. To date, no such approach has been proposed for NMP toxicity studies, with a number of less-than-ideal studies being published – resulting in an ever increasingly complicated landscape for risk assessors and regulators to navigate. For example, data reported from non-standard studies with limited attention towards QA/QC criteria and/or for which results are limited to monodisperse particles to which humans are not exposed, as represented in through the QA/QC evaluation reported in this study, represent particular challenges to risk assessors.

Tools such as the NMP-TSAT can also be used in helping to develop an understanding of potential toxicological mechanisms of action and in identifying potentially important research needs and data gaps. Ultimately, this information can also prove valuable in the context of developing AOPs for pathways relevant to NMPs.

Another important element to consider, relates to how screening and prioritization tools might be used to improve the communication of potential human health risks resulting from exposure to NMPs, whether it be from food and food packaging, drinking water and beverages or via inhalation of indoor or outdoor air. As NMPs have become a topic of public interest and media attention in recent years, an appreciation of the uncertainties inherent in the toxicity studies being conducted is imperative to ensuring that potential risks are accurately stated and appropriately reflect the current state-of-knowledge on the impacts to human health. Indeed, communicating the relevance of results associated with poorly characterized mondisperse polystyrene spheres in relation to sensitive endpoints are representative of challenges regulators currently face. Overall, the application of the tiered approach proposed in this study, which first screens and prioritizes toxicity studies based on their relative adequacy for reporting various QA/QC criteria, followed by expert evaluation regarding how the study was conducted and results reported, should help strengthen confidence in the decision-making process through a transparent and consistent approach aimed at characterizing and quantifying critical uncertainties. Decision-makers can then use the output of the tiered evaluation process to support risk assessment decisions and outcomes, to inform risk communication approaches and to robustly identify areas of research needed to reduce the relative level of uncertainty that may exist.

## Conclusion

In this study we develop and apply a screening and prioritization tool for evaluating a suite of QA/QC criteria reported in in vivo and in vitro effects studies aimed at characterizing and quantifying adverse effects of NMPs via inhalation or oral ingestion exposure pathways. A total of 74 studies were identified and evaluated using the tool developed and which represents a screening and prioritization tier 1 level of assessment. A total of 10 oral ingestion and 2 inhalation studies were prioritized for tier 2 – expert elicitation assessment. The results presented here complement other activities that have been initiated to support the Health Effects Workshop sponsored by the Southern California Coastal Water Research Project (SCCWRP) (https://www.sccwrp.org/about/research-areas/additional-research-areas/trash-pollution/microplastics-health-effects-webinar-series/. As a general observation, studies identified as following OECD guidance typically scored well with respect to study design and applicability for risk assessment, whereas a major shortcoming identified relates to the limited types of particles that have been studied. The majority of studies evaluated using the NMP-TSAT have been performed on monodisperse particles, predominately spheres (≈60% or 43/74), consisting of polystyrene (≈46% or 34/74). The other plastic polymers include a small number of studies that report results for polyethylene (11% or 8/74), PVC (5% or 4/74), polypropylene (3% or 2/74) and PET (3% or 2/74). Particle sizes are observed to be similar for both in vivo and in vitro effect studies, with the median particle size tested observed to be 2.2 μm and 0.5 μm, respectively. The majority of studies have tested particles < 5 μm, with a minimal particle size of 10 nm and a maximum particle size of about 200 μm. The challenge in applying the results of studies obtained from a suite of monodisperse particles to the heterogenous mixture of particles understood to represent environmentally relevant exposure is non-trivial and complicates the ability to reliably characterize and quantify the potential human health risks of NMPs.

Future research aimed at strengthening our understanding of the human health implications of NMPs will thus greatly benefit from:

- The generation of test materials representative of human exposure to NMPs for use in toxicity test systems, reducing the need to develop read-across methods to extrapolate results obtained from monodisperse particles to the complex heterogeneous mixture of environmentally relevant NMPs.
- Improved characterization and verification of test particle characteristics. In the absence of standard test materials representative of environmentally relevant NMPs, improved characterization of test particles would strengthen the correlation between observed adverse effects in both in vivo and in vitro test systems and various physicochemical properties of the particles tested.
- The adoption of study design guidance recommended by OECD when conducting either in vivo inhalation or oral ingestion toxicity tests. The adoption of OECD guidelines would greatly strengthen scoring against study design and application towards risk assessment criteria identified within the NMP-TSAT.

With an ever-increasing awareness and concern related to the potential for human health implications from exposure to NMPs, there is a critical need for determining the level of confidence in data produced by researchers and published in the peer-review literature. The development and application of the NMP-TSAT described in this study as part of a tiered-approach is perceived as an important contribution towards the acquisition of robust, reliable data, and will strengthen confidence in future decision-making processes.

## Supplementary Information

**Additional file 1: Table S1.** QA/QC scoring guidance against *in vivo* study criteria. **Table S2.** QA/QC scoring guidance against *in vitro* study criteria.

## Data Availability

The datasets used and analysed during the current study are available from the corresponding author on reasonable request.

## Abbreviations

AOP: Adverse outcome pathway
AT2: Primary human alveolar type 2 epithelial cells
BMD: Benchmark Dose
CRED: Criteria for reporting and evaluating ecotoxicity data
EFSA: European Food Safety Authority
JRC: Joint Research Centre
LDH: Lactate dehydrogenase assay
LOAEL: Lowest-observable adverse effect level
MAC: Primary Human alveolar macrophages
MPs: Microplastic particles
NMPs: Nano- and microplastic particles
NOAEL: No-observable adverse effect level
NPs: Nano-sized particles
OECD: Organisation for Economic Co-operation and Development
PET: Polyethylene terephthalate
PVC: Polyvinyl chloride
POD: Point of departure
QA/QC: Quality assurance / Quality Control
QIVIVE: Quantitative in vitro to in vivo extrapolation
ROS: Reactive oxygen species
SCCWRP: Southern California Coastal Water Research Project
TAS: Total Assessment Score
TSAT: Toxicity study assessment tool
TT1: A unique human alveolar type 1 epithelial cells
