# Supplementary material for: Screening and prioritization of nano- and microplastic particle toxicity studies for evaluating human health risks – development and application of a toxicity study assessment tool
Source: Microplast nanoplast. 2022 Jan 14;2(1):2. doi: 10.1186/s43591-021-00023-x (PMC8760192; doi:10.1186/s43591-021-00023-x)
Supplement: Supplementary file 1 — Additional file 1: Table S1. QA/QC scoring guidance against in vivo study criteria. Table S2. QA/QC scoring guidance against in vitro study criteria. [file 43591_2021_23_MOESM1_ESM.docx]

**Screening and prioritization of nano- and microplastic particle toxicity studies for evaluating human health risks – Development and application of a toxicity study assessment tool**

TODD GOUIN^*a^, Robert Ellis-Hutchings^b^, Leah Thornton Hampton^c^, Christine Lemieux,^d^ Stephanie Wright^e^

^a^ TG Environmental Research, Sharnbrook, Bedfordshire, UK

^b^ The Dow Chemical Company, Midland, MI 48673, USA

^c^ Department of Toxicology, Southern California Coastal Water Research Project, Costa Mesa, CA, USA.

^d^ Air Health Science Division, Water and Air Quality Bureau, Health Canada, Ottawa, ON, K1A 0K9, Canada

^e^ Environmental Research Group, School of Public Health, Imperial College London, Sir Michael Uren Hub, 86 Wood Lane, London W12 0BZ

To whom correspondence may be addressed:

^*^ Todd Gouin

TG Environmental Research

18 Wellpond Close

Sharnbrook, MK44 1PL

T: +44 1234 783548

[todd.gouin@environresearch.com](mailto:todd.gouin@environresearch.com)

**SUPPLEMENTARY INFORMATION**

**Table S1**: QA/QC scoring guidance against *in vivo* study criteria

| **Criteria** | **Suggested guidance for scoring studies** |
| --- | --- |
| **A: Particle Characterization** | |
| 1. Particle size | 1. Reported, but limited to average size as obtained from a supplier |
|  | 2. Reported, with specific information on particle size variance |
| 2. Particle shape | 1. Reported, but limited to average shape as obtained from a suppler |
|  | 2. Reported, with verification provided using high resolution images |
| 3. Polymer type | 1. Reported, but limited to composition obtained from supplier |
|  | 2. Reported, with verification using FTIR, Raman or other applicable approach |
| 4. Source of particles | 1. Reported, but limited to name of supplier/manufacturer |
|  | 2. Reported, with specific details related to how the particles were produced |
| 5. Particle surface chemistry | 1. Reported, but limited to information obtained from supplier |
|  | 2. Reported, with verification of the particle surface chemistry properties, such as charge, hydrophobicity, etc. in the test medium |
| 6. Chemical purity | 1. Reported, but limited to information obtained from supplier. No steps taken to clean particles or to remove chemical impurities, such as surfactants, stabilisers, emulsifiers, etc. |
|  | 2. Reported, with verification and/or evidence to demonstrate that particles appropriately cleaned |
| 7. Microbial contamination | 1. Reported as potentially present/absent. No steps taken to verify or remove |
|  | 2. Reported, with verification of the presence or absence of endotoxin. |
| **B: Experimental design** | |
| 1. Particle concentration units | 1. Reported, but limited to a single metric, such as mass/volume or number/volume |
|  | 2. Reported with details provided for both mass/volume and number/volume |
| 2. Particle stability ^a^ | 1. Studies that provide limited (qualitative/semi-quantitative) information supporting particle stability within the test medium as assessed by the evaluator. |
|  | 2. Studies that measure and verify particle stability within the test medium, inlcuding details of aggregation kinetics and/or high resolution digital images, or other method assessed as appropriate by the evaluator, such as characterization of zeta-potentials and behaviour in water versus medium. |
| 3. Test medium and/or delivery vehicle | 1. Partial inclusion of information, such as when more than one test condition is used. |
|  | 2. Test medium or vehicle used to dose particles is fully reported. |
| 4. Administered dose/concentration | 1. Nominal test doses or concentrations are reported in the exposure media, with dilution factors. |
|  | 2. Test doses or concentrations in the exposure media, with dilution factors are reported, which are verified analytically, i.e. represent the actual dose or concentration. |
| 5. Homogeneity of exposure | 1. Limited information pertaining to the homogeneity of the exposure dose is reported and which is defined as insufficient with explanation by the evaluator. Aqueous/intratacheal/intrapertioneal solutions of MPs administered need to be representative of well-mixed or dispersed in solutions, dietary exposure requires evidence of uniform distribution throughout the food item, data supporting the homogeneity of aerosols used for nose-only inhalation studies. |
|  | 2. Verification pertaining to the homogeneity of the exposure dosed. Aqueous/intratacheal/intrapertioneal solutions of MPs administered need to be representative of well-mixed or dispersed in solutions, dietary exposure requires evidence of uniform distribution throughout the food item, data supporting the homogeneity of aerosols used for nose-only inhalation studies. |
| 6. Administration route | 1. Limited Specifications reported on how test particles were administered to test animals and which is defined as being insufficient with explanation by the evaluator. Examples include:  - dilution of the test item in diet, in vehicle, solvent,  - total volume applied by gavage,  - preparation of aerosol or atmospheres in inhalation studies,  - handling of animals under treatment (e.g. in nose-only studies),  - type of occlusion and exposed skin area in dermal exposure studies |
|  | 2. Specifications reported on how test particles were administered to test animals. Examples include:  - dilution of the test item in diet, in vehicle, solvent,  - total volume applied by gavage,  - preparation of aerosol or atmospheres in inhalation studies,  - handling of animals under treatment (e.g. in nose-only studies),  - type of occlusion and exposed skin area in dermal exposure studies |
| 7. Test species | 1. Limited information reported related to the test species, such as name, age, sex, body weight and information on the strain of the test animals and which is defined as insufficient with explanation by the evaluator. |
|  | 2. All details related to the test species, such as name, age, sex, body weight and information on the strain of the test animals is reported. At the commencement of the study the weight variation of animals used should be minimal and not be > 20% of the mean weight of each gender |
| 8. Feeding/housing conditions | 1. Limited information reported regarding the specifications of the feeding and housing of test animals and which is defined as insufficient by the evaluator with explanation. |
|  | 2. Satisfactory description of the specifications reported regarding the feeding and housing of test animals |
| 9. Sample size | 1. Number of individuals per test group reported. Depending on the animal model used and experimental design the sample size is defined as insufficient by the evaluator with explanation. |
|  | 2. Satisfactory reporting of the numbers of individuals per test group reported. As a guide the respective repeat-dose OECD 407 or 408 requires at least 10 animals (five female and five male) or 20 animals (ten female and ten male) should be used at each dose level. This sample size and gender representation is required for a score of '2' |
| 10. Frequency and duration of exposure | 1. Insufficient information reported to fully evaluate the frequency and duration of the exposure, as well as time-points or concerns raised regarding the relevance of the duration of the exposure with evaluator explanation. |
|  | 2. Satisfactory reporting on the frequency and duration of the exposure, as well as time-points of observations. |
| 11. Controls | 1. Study includes a negative/vehicle control |
|  | 2. Study includes the reporting of a particle and/or positive control |
| 12. Treatment groups | 1. Study reports the inclusion of at least 3 separate treatment exposure groups but an insufficient sample size |
|  | 2. Study includes the inclusion of at least 3 separate treatment exposure groups and meeting the minimum required sample size for repeat oral dose experiments |
| 13. Confirmation of internal dose | 1. Insufficient information reporting verification that >80% of the nominal concentration is bioavailable supported by evaluator explanation, or that staining is used to identify and quantify particles |
|  | 2. Verification that >80% of the nominal concentration is bioavailable. Exposure verified and quantified by FTIR or Raman or other appropriate analytical tool |
| **C. Applicability for risk assessment** | |
| 1. Statistical analysis | 1. Statistical methods should be suitable for the dataset under analysis and the goal of the analysis. In case statistical methods are provided but the evaluator is not able to judge the suitability of the statistical methods, this question should be scored by 1 with explanation. |
|  | 2. Statistical methods should be suitable for the dataset under analysis and the goal of the analysis. |
| 2. Endpoints ^b^ | 1. Sub-organismal level endpoints, such as biomarkers, are used and endpoints sufficient to assess response as either adaptive or adverse effect at higher level of biological organization |
|  | 2. Individual level effects, such as growth or reproduction, are reported, or sub-organismal level endpoints obtained from biomarkers are extrapolated with relevance to human health |
| 3. Dose-response relationship ^c^ | 1. Dose-response relationships based on ≥3 exposure concentrations with a concentration range ≥ 2.5x, excluding the control. |
|  | 2. Dose-response relationships based on ≥3 exposure concentrations with a concentration range ≥2.5x, excluding the control. Is there a concentration (or other particle descriptor) dependent response? Score '2' if reasonable explanation given to interpret dose-response relationship. |
| 4. Concentration range ^d^ | 1. Unable to demonstrate environmentally relevant concentration range, supported with arguments. |
|  | 2. Concentration range is reported to be consistent with environmentally relevant concentrations. |
| 5. Effect threshold | 1. Effect thresholds reported as NOEC/NOAEL, LOEC, or when no error data are provided. Alternatively, if no thresholds reported, the study provides access to raw data to enable threshold values to be derived. A descending sequence of dose levels should demonstrate a dose-related response and a NOAEL at the lowest dose level |
|  | 2. Effect threshold concentrations, accompanied with estimates of error or uncertainty, and which reflect the L(E)Cx dervied from dose-response relationship modelling, with error data (95% confidence interval, standard error or standard deviation). |
| 6. Test particle relevance^e^ | 1. The diversity of the particles is limited to assessing only one or two properties, such as looking at different particle sizes, shapes, surface charge or densities of the same polymer or comparing between two different polymers of same properties. |
|  | 2. Studies that use multiple types of particles and combinations of properties that reflect a variety of sizes, shapes, surface charge and densities in one mixture exposure. |

Assign a score of zero (0) if:

^a^ only nominal concentration reported.

^b^ not reported or insufficient to assess if endpoint relates to adaptive or adverse effect.

^c^ not reported or uses ≤2 exposure concentrations.

^d^ tested concentration range much greater (i.e. >1000x) than the range of environmentally relevant concentrations.

^e^ Not reported or only limited to a single type of particle and single property.

**Table S2**: QA/QC scoring guidance against *in vitro* study criteria

| **Criteria** | **Suggested guidance for scoring studies** |
| --- | --- |
| **A: Particle Characterization** | |
| 1. Particle size | 1. Reported, but limited to average size as obtained from a supplier |
|  | 2. Reported, with specific information on particle size variance |
| 2. Particle shape | 1. Reported, but limited to average shape as obtained from a suppler |
|  | 2. Reported, with verification provided using high resolution digital images |
| 3. Polymer type | 1. Reported, but limited to composition obtained from supplier |
|  | 2. Reported, with verification using FTIR, Raman or other applicable approach |
| 4. Source of particles | 1. Reported, but limited to name of supplier/manufacturer |
|  | 2. Reported, with specific details related to how the particles were produced |
| 5. Particle surface chemistry | 1. Reported, but limited to information obtained from supplier |
|  | 2. Reported, with verification of the particle surface chemistry properties, such as charge, hydrophobicity, etc. in the test medium |
| 6. Chemical purity | 1. Reported, but limited to information obtained from supplier. No steps taken to clean particles or to remove chemical impurities, such as surfactants, stabilisers, emulsifiers, etc. |
|  | 2. Reported, with verification and/or evidence to demonstrate that particles appropriately cleaned |
| 7. Microbial contamination | 1. Reported as potentially present/absent. No steps taken to verify or remove |
|  | 2. Reported, with verification of the presence or absence of endotoxin. |
| **B: Experimental design** | |
| 1. Particle concentration units | 1. Reported, but limited to a single metric, such as mass/volume or number/volume |
|  | 2. Reported with details provided for both mass/volume and number/volume |
| 2. Particle stability ^a^ | 1. Studies that provide limited (qualitative/semi-quantitative) information supporting particle stability within the test medium as assessed by the evaluator. |
|  | 2. Studies that measure and verify particle stability within the test medium, inlcuding details of aggregation kinetics and/or high resolution digital images, or other method assessed as appropriate by the evaluator, such as characterization of zeta-potentials and behaviour in water versus medium. |
| 3. Test medium and/or delivery vehicle | 1. Partial inclusion of information, such as when more than one test condition is used. |
|  | 2. Test medium or vehicle used to dose particles is fully reported. |
| 4. Applied dose/concentration | 1. Nominal test doses or concentrations are reported in the exposure media, with dilution factors. |
|  | 2. Test doses or concentrations in the exposure media, with dilution factors are reported, which are verified analytically, i.e. represent the actual dose or concentration. |
| 5. Homogeneity of exposure | 1. Limited information pertaining to the homogeneity of the exposure dose is reported and which is defined as insufficient with explanation by the evaluator. MPs administered should ideally be representative of well-mixed or dispersed in solutions within the in vitro test system. |
|  | 2. Verification pertaining to the homogeneity of the exposure dosed. MPs administered are demonstrated to be well-mixed or dispersed within the in vitro test system. Characterization of polydispersity index (PDI) may represent a substitute to support an understanding that particles have a high homogeneity in the particle population (i.e. PDI <0.2 strengthens assumption of high homogeneity) |
| 6. Description of fundamental in vitro model test system elements | 1. Description must include, for example, type of cells or tissue used: primary cells, cell lines, reconstructed tissue, isolated (parts of) organs, bacteria, yeast cells. Assign a value of '1' if origin/source of test system not described, examples for such information are:  - laboratory/scientist providing cell lines,  - commercial provider of test systems,  - origin of ex vivo organs, tissues, primary cells etc. |
|  | 2. Description may include, for example, type of cells or tissue used: primary cells, cell lines, reconstructed tissue, isolated (parts of) organs, bacteria, yeast cells. A value of '2' assigned where description of source/origin of test system is included. |
| 7. Inclusion of additional test system parameters | 1. Limited information providing additional important information: eg. Cell density used, sample volume, well surface, vehicle or solvent used; maximum concentration of solvent; explanation of specific conditions applying during exposure (static, dynamic, with light, darkness...); application of new medium on or after exposure; limited information describing how endpoints were determined (cytotoxicity, ROS generation, misfolded proteins, cytokines, etc) |
|  | 2. Exceptional details provided describing the method: eg. Cell density used, sample volume, well surface, vehicle or solvent used; maximum concentration of solvent; explanation of specific conditions applying during exposure (static, dynamic, with light, darkness...); application of new medium on or after exposure; Detailed information describing how endpoints were determined (cytotoxicity, ROS generation, misfolded proteins, cytokines, etc) |
| 8. Sample size / Replicates (e.g. min 10^6^ cells/well; 2-3 replicates) | 1. Depending on the in vitro test system model used and experimental design the sample size is defined as insufficient by the evaluator with explanation. |
|  | 2. Satisfactory reporting of the sample size / replicates. |
| 9. Frequency and duration of exposure | 1. Insufficient information reported to fully evaluate the frequency and duration of the exposure, as well as time-points or concerns raised regarding the relevance of the duration of the exposure with evaluator explanation. |
|  | 2. Satisfactory reporting on the frequency and duration of the exposure, as well as time-points of observations. Time points of observations may not be mentioned when the experimental set-up makes clear that observation takes place immediately after end of exposure (considered sufficient). Please check also figures and tables for respective information. |
| 10. Controls | 1. Study includes a negative/vehicle control |
|  | 2. Study includes the reporting of a particle and/or positive control |
| **C. Applicability for risk assessment** | |
| 1. Statistical analysis | 1. Statistical methods should be suitable for the dataset under analysis and the goal of the analysis. In case statistical methods are provided but the evaluator is not able to judge the suitability of the statistical methods, this question should be scored by 1 with explanation. |
|  | 2. Statistical methods should be suitable for the dataset under analysis and the goal of the analysis. |
| 2. Endpoints ^b^ | 1. Endpoints insufficient to assess if endpoint relates to an adaptive or adverse effect in humans |
|  | 2. Endpoints sufficient to assess response as either adaptive or adverse effect in humans |
| 3. Dose-response relationship ^c^ | 1. Dose-response relationships based on ≥3 exposure concentrations with a concentration range ≥ 2.5x, excluding the control. |
|  | 2. Dose-response relationships based on ≥3 exposure concentrations with a concentration range ≥2.5x, excluding the control. Is there a concentration (or other particle descriptor) dependent response? Score '2' if reasonable explanation given to interpret dose-response relationship. |
| 4. Concentration range ^d^ | 1. Unable to demonstrate environmentally relevant concentration range, supported with arguments. |
|  | 2. Concentration range is reported to be consistent with environmentally relevant concentrations. |
| 5. Effect threshold | 1. Effect thresholds reported as No observed effect concentration/no observed adverse effect level (NOEC/NOAEL), lowest observed effect concentration (LOEC), or when no error data are provided. Alternatively, if no thresholds reported, the study provides access to raw data to enable threshold values to be derived. |
|  | 2. Effect threshold concentrations, accompanied with estimates of error or uncertainty, and which reflect the L(E)Cx dervied from dose-response relationship modelling, with error data (95% confidence interval, standard error or standard deviation). |
| 6. Test particle relevance^e^ | 1. The diversity of the particles is limited to assessing only one or two properties, such as looking at different particle sizes, shapes, surface charge or densities of the same polymer or comparing between two different polymers of same properties. |
|  | 2. Studies that use multiple types of particles and combinations of properties that reflect a variety of sizes, shapes, surface charge and densities in one mixture exposure. |

Assign a score of zero (0) if:

^a^ only nominal concentration reported.

^b^ not reported or insufficient to assess if endpoint relates to adaptive or adverse effect.

^c^ not reported or uses ≤2 exposure concentrations.

^d^ concentration range much greater than the range of environmentally relevant concentrations.

^e^ Not reported or only limited to a single type of particle and single property.
